# Disruptions and General Distress for Essential and Nonessential Employees During the COVID-19 Pandemic

**DOI:** 10.1007/s10869-021-09744-5

**Published:** 2021-04-01

**Authors:** Ward van Zoonen, Claartje L. ter Hoeven

**Affiliations:** 1 Amsterdam School of Communication Research, University of Amsterdam, Nieuwe Achtergracht 166, 1018 WV Amsterdam, The Netherlands; 2 Erasmus School of Social and Behavioral Sciences (ESSB), Erasmus University Rotterdam, Burgemeester Oudlaan 50, 3062 PA Rotterdam, The Netherlands

**Keywords:** Work stressors, Social stressors, General distress, COVID-19, Essential and nonessential work, Change communication quality

## Abstract

The COVID-19 pandemic and outbreak response represent a global crisis that has affected various aspects of people’s lives, including work. Speculation is rife about the impact of the crisis on employees. Countries and organizations worldwide have categorized some work as essential and, by extension, other work as nonessential. This study aims to investigate the impact of the pandemic by examining the relationship between work disruptions (at time 1) and general distress (at time 2) through various work stressors, contrasting the experiences of employees in essential versus nonessential work. For employees with essential jobs, there is a significant indirect effect of work disruptions on general distress through hindrance stressors. This relationship is not found for employees with nonessential jobs. The general distress of these employees is more strongly affected by disruptions through social stressors (here, social isolation). Hence, this study demonstrates how general distress is affected in different ways for employees conducting essential work and those conducting nonessential work. We further highlight the importance of considering social stressors in this relationship, especially for nonessential work. Organizational change communication quality mitigates the relationship between isolation and general distress for employees with nonessential jobs, but not for those with essential jobs.

## Introduction

The COVID-19 pandemic has had a profound impact on many aspects of the society, including work (Carnevale & Hatak, 2020; Gibson, 2020; Kramer & Kramer, 2020; Rudolph et al., 2020; Spurk & Straub, 2020) and mental health (Holmes et al., 2020; Sinclair et al., 2020). For instance, the crisis has vividly highlighted the extent to which the society relies on the so-called essential work (Guasti, 2020) and, by extension, has explicitly designated much work as nonessential. Definitions of what essential work entails tend to vary. Historically, definitions of essential workers have focused on medical personnel (Matsuishi et al., 2012) and have been applied more broadly to include police and other first responders (Gershon et al., 2010). During the COVID-19 pandemic, the definitions of what essential work entails expanded to include postal delivery, food distribution, and internet and communication services (Benhamou & Piedra, 2020). The definitions used by the Dutch government, and many others, describe essential work as all work that is crucial to keep the economy and society running (Kulow et al., 2020), including “care; youth aid and social support; … transportation and production of medicine and medical devices; teachers and school staff, required for online learning, exams and childcare; public transportation; food production and distribution, such as supermarkets, food production and food transportation, farmers, and farmworkers; transportation of fuel, coal, diesel, and so forth; transportation of waste and garbage; daycare; media and communications; emergency services such as fire department, ambulance, and regional medical organizations; and necessary administrative services on the provincial and municipal levels. In addition, about 100 companies have been identified as necessary to sustain public life, operating in sectors such as gas and fuel production, distribution and transportation, communication and online services, water supply, securities trading, and infrastructure.” (von Gaudecker et al., 2020, p. 4-5).

The COVID-19 measures in the Netherlands and elsewhere have included specific instructions related to specific work. Governments worldwide have created lists of essential work and processes.^1^ Workers conducting this work are designated essential workers who are allowed, or urged, to go to work to keep the society running (Guasti, 2020) (these essential workers represent approximately 33% of the Dutch workforce).^2^ All other employees are expected to work from home or are simply requested to stay inside. We seek to explore three underlying mechanisms to help understand how work disruptions triggered by the COVID-19 pandemic relate to general distress, differentiating between stressors in essential and nonessential work. In addition to the potential transmission of the virus, major concerns about isolation, job demands, and insecurity caused by the COVID-19 pandemic and outbreak responses may have substantial and long-lasting impacts on mental health (Campion et al., 2020; Daly & Robinson, 2020; Holmes et al., 2020). Hence, a constellation of factors may contribute to worsening mental health, particularly increasing distress (Holmes et al., 2020). Distress is a state of emotional suffering associated with stressors and demands that are difficult to cope with (Arvidsdotter et al., 2016). Specifically, work-related factors—such as high demands, poor support, and lack of control—have been identified as factors that contribute to distress (Marchand et al., 2005). Distress is a common mental health problem, and, especially during the COVID-19 pandemic, those experiencing mental health problems may not have access to the necessary help (Daly & Robinson, 2020). Though this study is not concerned with the diagnosis or treatment of mental health problems, we seek to identify which occupational groups (essential versus nonessential) may be particularly vulnerable to experiencing mental health problems and to identify the underlying difficulties that contribute to these problems.

Extreme events have often been recognized by researchers as opportunities to uncover unique dynamics and information that might otherwise remain hidden (Hällgren et al., 2018). The COVID-19 pandemic constitutes such an extreme event. In its most basic form, “an event reflects a discrete, discontinuous happening that diverges from the stable or routine features of the organizational environment” (Morgeson, et al., p. 519). Extreme events may exceed an organization’s capacity to prevent or control certain outcomes and cause massive psychological consequences that can be considered distressing by organizational members (Hällgren et al., 2018; Hannah et al., 2009, p. 898). These events can be seen as causing a number of occurrences over time or downstream in organizations (Morgeson et al., 2015). The COVID-19 pandemic, for instance, has caused a number of disruptions in work procedures, as outbreak response strategies have forced social-distancing practices and remote work mandates to be implemented (Gibson, 2020; Rudolph et al., 2020; Spurk & Straub, 2020). Hence, we investigate COVID-19-related disruptions—e.g., changes in work procedures, information sharing, and coordination of work—and how these disruptions trigger different stressors in essential versus nonessential work.

To investigate the work disruption-general distress relationship, we draw on the challenge stressor-hindrance stressor framework (e.g., Cavanaugh et al., 2000; LePine et al., 2005; Rodell & Judge, 2009). Challenge stressors refer to job demands that are challenging yet can be rewarding by creating opportunities for personal growth and excellence (Cavanaugh et al., 2000; Rodell & Judge, 2009). Hindrance stressors are demands that are viewed as obstacles that obstruct personal growth and hinder one’s ability to achieve desired goals (Cavanaugh et al., 2000; Rodell & Judge, 2009). We suggest that hindrance and challenge stressors are especially exacerbated for employees with essential jobs and include job responsibilities, time pressure and workload (i.e., challenge stressors), as well as the potential ambiguity and regulations or red tape one needs to manage (i.e., hindrance stressors). For instance, during the pandemic, there has been pressure on intensive care units, where the number of patients has pushed the limits of our health care systems, and the perceived responsibility of the employees in these units has increased as the eyes of the world are fixed on the health care professionals we rely on. Another example is law enforcement, who suddenly have had to police complex and ambiguous regulations.^3^ Many of these pandemic-related problems seem less profound for employees with nonessential jobs, as they have been requested to continue their work mostly as they did before but from a remote location. Arguably, this situation has triggered changes in information sharing, organizing, and coordinating work; however, these changes may be less consequential in terms of challenge and hindrance stress appraisals. Therefore, we expect that these stressors operate as two important underlying mechanisms in the relationship between work disruptions and general distress, especially for employees in essential rather than nonessential jobs.

Cognizant of social-distancing and remote work mandates, we extend our investigation to include social stressors (Dormann & Zapf, 2004; Harris et al., 2009). Specifically, we consider social isolation as a third underlying mechanism in the relationship between disruption and general distress. Social distancing is one of the focal points in the outbreak response strategy and has been found to negatively impact (employee) well-being (Brooks et al., 2020; Venkatesh & Edirappuli, 2020). Isolation has been identified as a major challenge for employees during the COVID-19 pandemic (Hamouche, 2020) and earlier health pandemics (Siu, 2008). Missing the possibility to interact and connect face-to-face with colleagues can increase employees’ sense of isolation (Cooper & Kurland, 2002; Marshall et al., 2007; Tavares, 2017). Isolation is assumed to be especially profound for employees in nonessential jobs working under remote work directives compared to employees in essential jobs, as employees in nonessential jobs are deprived of any interactions with their colleagues in close proximity. Last, we argue that the provision of useful, timely, and accurate information on the crisis and its consequences for work by organizations (Bordia et al., 2004) can mitigate the negative impact of work and social stressors on general distress, as the provision of such information equips employees with the necessary information to deal with these stressors.

Hence, this study examines the impact of work disruptions during the COVID-19 pandemic on general distress by examining work and social stressors, as well as by investigating the potential buffering impact of organizational communication quality. In doing so, we seek to make at least three contributions. First, we contribute to the extreme context research by investigating how acute work disruptions triggered by extreme events (Hällgren et al., 2018; Morgeson et al., 2015) relate to work stressors (Cavanaugh et al., 2000; Rodell & Judge, 2009), social stressors (Dormann & Zapf, 2004) and ultimately mental health at the individual level (Cavanaugh et al., 2000; LePine et al., 2005; Rodell & Judge, 2009). Second, we expect to find different underlying mechanisms in the relationship between work disruptions and general distress for employees in essential versus nonessential jobs. By explicitly considering social stressors as part of the challenge stressor-hindrance stressor framework (Cavanaugh et al., 2000; Dormann & Zapf, 2004), we hypothesize that employees in essential jobs experience general distress through work stressors (e.g., time pressures and ambiguity), whereas employees in nonessential jobs experience general distress through social stressors (i.e., social isolation). Finally, this study offers a more practical contribution by examining the influence of the quality of change communication regarding the COVID-19 pandemic provided by the organization. The findings of this study are important as they demonstrate that organizations should not only be wary of extraorganizational events as sources of workplace stressors but also recognize that different groups and individuals are impacted differently. Doing so requires various (organizational) interventions for different occupational groups and employees.

## Theory

### Disruptive Events and General Distress

Events play a major role in how we think, feel, and act. In an organizational context, events occur at every hierarchical level from the molar environmental level to the molecular individual level (Morgeson et al., 2015). Extraorganizational events can trigger change processes and disrupt work routines (Weick & Quinn, 1999); therefore, these events may increase occupational stress (Cooper & Davidson, 1987). Various models of stress and coping provide insights into how individual well-being is affected by different types of threats, including major events, threatening situations, and daily hassles (Dewe et al., 2010; Lazarus & Folkman, 1984). Indeed, it has long been recognized that various situations outside the direct work environment can trigger stressors at work (Beal & Ghandour, 2011). Especially in the context of essential workers, e.g., police and nurses, the impact of extraorganizational events on job stressors has been investigated (Evans, 2002; Garbarino et al., 2011). Hence, we assume that disruptions triggered by the COVID-19 pandemic increase various work and social stressors.

### Job Stressors and General Distress

Drawing on the job stressor literature, we differentiate between work stressors and social stressors (Cavanaugh et al., 2000; Dormann & Zapf, 2004). Specifically, we assume that different stressors play different roles for employees in essential versus nonessential jobs. Employees in essential jobs are requested to go to work and keep the society running; however, daily work practices may be disrupted by increased constraints, work hours, regulations, and procedures. These disruptions may exacerbate experienced time pressure, workload, and responsibilities, and they may increase the amount of red tape these employees must manage and the amount of uncertainty they experience due to new (safety) regulations. These demands are typically categorized as challenge and hindrance stressors (Cavanaugh et al., 2000; Crawford et al., 2010; Rodell & Judge, 2009). Based on Cavanaugh et al. (2000), we suggest that challenge stressors can be viewed as job demands that can be rewarding, as they may also present opportunities for personal growth (Prem et al., 2017). Examples of challenge demands are workload, job complexity and job responsibility (Rodell & Judge, 2009). These challenges are appraised as stressful but have the potential to promote mastery, growth, and future gains (Crawford et al., 2010). Hindrance stressors are job demands that are primarily viewed as barriers or obstacles for personal growth and development and may hinder individuals in achieving desired goals (Rodell & Judge, 2009). Examples of hindrance stressors are ambiguity, role conflict, and red tape. These hindrances tend to be appraised as stressful and have the potential to thwart growth and goal attainment (Crawford et al., 2010).

Although several studies suggest that hindrance *and* challenge stressors are positively related to strain (Crawford et al., 2010; LePine et al., 2005; Podsakoff et al., 2007; Rodell & Judge, 2009), challenge stressors seem to operate in more ambivalent terms by also producing favorable effects, such as enhanced motivation, resilience, and performance (Crane & Searle, 2016; LePine et al., 2005; Podsakoff et al., 2007; Prem et al., 2017). van den Broeck et al. (2010) aptly describe this difference as follows: “Job hindrances are threatening constraints, which deplete employees’ energy and elicit an emotion-focused coping style. […] they are likely to relate negatively to employees’ optimal functioning in terms of well-being, favorable attitudes, and constructive behavior. Job challenges, in contrast, are obstacles that can be overcome. They require energy, but are simultaneously stimulating” (p. 741). Several empirical studies corroborate the differential workings of hindrance and challenge stressors with regard to well-being. For example, in a longitudinal study, Crane and Searle (2016) found that challenge stressors can be important for building resilience, which reduces psychological strain. van den Broeck et al. (2010) replicated a positive relationship between challenge stressors and vigor at work across two different samples. Similarly, Prem et al. (2017) demonstrated that challenge stressors were related to thriving at work, i.e., learning, not vitality, which could improve employees’ health (Porath et al., 2012). Hence, in line with Kern et al. (2020)—who suggested that challenge stressors can increase performance, engagement and well-being—we suggest that challenge stressors may reduce distress because they are related to the attainment of personal work goals (van den Broeck et al., 2010), have a fair chance of being managed successfully (Pearsall et al., 2009), offer opportunities for personal gains and growth (Crane & Searle, 2016; Prem et al., 2017), and can be viewed as a legitimate aspect of the job that is either inherent to the work tasks or difficult to avoid under the circumstances of the job (Kern et al., 2020). Conversely, hindrance stressors typically do not offer any potential gains, can be viewed as unnecessary or unreasonable, and lead more univocally to psychological strain (Kern et al., 2020), while challenge stressors could have positive implications on well-being.

Within the body of literature about challenge and hindrance stressors, several studies point toward the importance of the conditions or contextual factors, such as job roles and occupations, in stressor appraisal and effects (Bakker & Sanz-Vergel, 2013; Naseer et al., 2020; Webster et al., 2011). Johns (2006) proposed considering two levels of analysis regarding context: omnibus contextual factors, such as occupation and time, and discrete contextual factors, such as task and social factors. The COVID-19 pandemic triggered omnibus contextual factors by categorizing jobs as either essential or nonessential. This categorization, we argue in line with Johns (2006), implies which discrete contextual variables provide an explanatory link between more descriptive and general omnibus contexts and specific work outcomes. Thus, we argue that event-induced disruptions trigger challenge and hindrance stressors differently for employees in essential and nonessential jobs. More specifically, in the context of the COVID-19 pandemic, reliance on employees with essential jobs is likely to increase their challenge stressors, such as additional responsibilities and increased job complexity, and their hindrance stressors, such as new safety regulations and increased task ambiguity. For example, during the pandemic, law enforcement and health care both come with new responsibilities for managing public health (challenge stressors) *and* new safety regulations, such as social distance regulations and other health precautions (hindrance stressors). Meanwhile employees with nonessential jobs are asked to sit out the storm and work from home, which may not increase responsibilities and regulations to the same degree (if at all). Therefore, we hypothesize that the relationship between work disruptions and general distress through challenge stressors and hindrance stressors will be more pronounced for essential workers compared to nonessential workers:

1. Occupational group moderates the negative indirect relationship between perceived disruptions and general distress through increased challenge stressors, such that this relationship is stronger for those in essential compared to those in nonessential work.
2. Occupational group moderates the positive indirect relationship between perceived disruptions and general distress through hindrance stressors, such that this relationship is stronger for those in essential compared to those in nonessential work.

### Workplace Isolation and General Distress

Workplace isolation refers to employees’ perceptions of isolation from others at work and includes perceived isolation from both colleagues and the company’s support network (Marshall et al., 2007); this isolation is considered a social stressor. Social stressors are defined as a class of characteristics, situations, episodes, or behaviors that are related to psychological or physical strain and that are in some way social in nature (Dormann & Zapf, 2004, p. 62; Harris et al., 2009). Other examples of social stressors include poor group climate or coworker conflict. From an evolutionary perspective, isolation typically triggers alarm in humans, which they respond to by seeking the company of others (Bowlby, 1973). However, opportunities for interaction, networking, and support diminish when employees are located in dispersed locations or work under social-distancing regulations. The sudden separation from the workplace and colleagues might trigger feelings of isolation and loneliness, especially for employees conducting nonessential work. Hence, we specifically consider isolation as a social stressor underlying the relationship between disruptions and general distress.

Research has suggested that remote work may deprive employees of organizational support networks (Collins et al., 2016). This deprivation may trigger perceptions of isolation, which have been found to be a major issue for employees in remote offices (Cooper & Kurland, 2002; Marshall et al., 2007). Social isolation is found to affect employees’ mental health not only in remote work contexts but also among flight attendants (Chen & Kao, 2012) and in the context of workplace bullying, where the victim may feel (socially) isolated (Einarsen & Nielsen, 2015). The nature of current work disruptions may trigger social isolation due to physical and social separation, which may lead to perceptions of a lack of available support, missed opportunities for (in)formal interactions with coworkers, and a feeling of not being part of the workgroup. Previous studies have demonstrated that perceptions of isolation contribute to individual problems, including depression (Rich & Scovel, 1987) and psychological distress (Nelson & Quick, 1985), both in the workplace and beyond, as perceived social isolation has been found to have negative effects on mental health (Novak et al., 2011). As such, we argue that the work disruptions caused by the COVID-19 pandemic will trigger social stressors. Employees with nonessential jobs are those who are most clearly separated from their colleagues and office spaces. Employee with essential jobs, even though maintaining a six-foot distance, often still work at their primary work locations in (closer) proximity to coworkers. Hence, we hypothesize that work disruptions trigger social stressors primarily for nonessential rather than for essential workers, thereby increasing general distress:

- H2: Occupational group moderates the positive indirect relationship between perceived disruptions and general distress through workplace isolation, such that this relationship is stronger for those in nonessential compared to those in essential work.

### The Moderating Role of Change Communication Quality

Research has examined various conditions under which stressor-strain relationships may be more or less profound, including the role of perceived organizational support (Wallace et al., 2009), charismatic leadership (LePine et al., 2016), and leader-member exchange (Montani et al., 2017). Arguments to support these moderating relationships are often based on the premise that “support may serve an informational purpose that helps employees functionally cope with stressors” (Wallace et al., 2009, p. 794). Similarly, the role of charismatic leadership in the stressor-outcome relationship demonstrates that leaders’ behaviors, including the communication of optimism (LePine et al., 2016, p. 1044), may help to attenuate the negative consequences of work stressors. Having timely access to adequate information may increase the feeling of having control over a situation and the resources to minimize the effects of stressors (Wallace et al., 2009).

High-quality communication provides information not only about work but also about the wider work environment (Parker et al., 2001). Parker et al. (2001) argued that good-quality communication is a sign of organizational support that leads employees to direct their efforts and orientations toward collective organizational goals. Research has long recognized the role of communication in providing work and social support. For instance, according to Cobb (1976), social support can be viewed as “information that leads individuals to believe that they are A) cared for and loved, B) esteemed and valued and C) that they participate in a network of communication and mutual obligation” (p. 300).

During inherently uncertain times, employees are more likely to look to their leaders for guidance and support (Waldman et al., 2001). Therefore, the relationship between stressors and strain may be conditional upon the extent to which their organization is communicating in ways that are useful to them, that provide them with answers, that are positive, and that are appropriate for the situation (Bordia et al., 2004; LePine et al., 2016). By offering accurate information and support, organizations provide employees with resources to better deal with the challenges and hindrances they face (Wallace et al., 2009). Provided employees are expected to work, regardless of the category or occupation, job conditions, like challenge and hindrance demands, will apply. Therefore, we expect a moderating effect of the quality of change communication between stressors and general distress for both essential and nonessential workers. Regarding challenge stressors, this expectation means that the assumed negative association with general distress will be strengthened by quality communication. The hypothesized positive relationship between hindrance stressors and general distress will presumably be diminished by the experience of high-quality communication. The same interaction effect of quality communication is expected in the relationship between workplace isolation and general distress. We believe this to be true for both essential *and* nonessential workers, since both groups must deal with social distancing and seeing (some) colleagues less often (Gibson, 2020). Based on this reasoning, we propose the following:

- H3: The relationship between challenge stressors and general distress is moderated by communication quality, such that higher (vs. lower) communication quality increases the negative relationship between challenge stressors and general distress for both employees conducting essential and nonessential work.
- H4: The relationship between hindrance stressors and general distress is moderated by communication quality, such that higher (vs. lower) communication quality reduces the positive relationship between hindrance stressors and general distress for both employees conducting essential and nonessential work.
- H5: The relationship between workplace isolation and general distress is moderated by communication quality, such that higher (vs. lower) communication quality reduces the positive effect of workplace isolation on general distress for both employees conducting essential and nonessential work.

## Methods

### Sample

We drew on a sample of 313 Dutch employees: 166 conducted nonessential work, and 147 conducted essential work. We tasked Dynata, a market research firm, with recruiting the respondents. Dynata engaged in diverse and quota-sampling techniques to obtain representative samples (e.g., age and gender) of essential and nonessential workers from various panels with double opt-in panelists to ensure engaged, reliable, and diverse responses. The professions and profiles of panelists were cross-referenced with the list of essential work and vital processes published by the government.^4^ Furthermore, respondents qualified for participation based on several screening questions, including those regarding the respondents’ work sectors, work hours, and ages. We asked respondents to indicate whether they worked in an essential sector and whether they worked on crucial processes as defined by the government. If so, respondents were prompted to indicate which sector and processes their work entailed. This procedure led to a categorization of essential and nonessential workers. Finally, only respondents that passed the attention and quality checks were retained; others were screened out.

We asked respondents about perceived disruptions, as well as work and social stressors in the first survey, and contacted the respondents again approximately 3 weeks later with a follow-up survey asking about their mental health. Both surveys (Time 1 (T1), April 8–April 14, 2020; Time 2 (T2), May 4–May 15, 2020) were conducted during the COVID-19 crisis while the country was in partial lockdown. The lockdown measures were announced on March 16, 2020 and the first relaxation of the measures was announced on May 16, 2020, after data collection was finished. During the lockdown measures, schools were closed (except for those children whose parents worked in essential professions), and the government announced a remote work mandate for all nonessential personnel.

The respondents with essential jobs primarily worked in health care (49.7%, *n* = 73), while others worked in food supply (15%, *n* = 22) and essential government processes (8.8%, *n* = 13). Respondents with nonessential jobs worked predominantly in the trade and professional service sectors (14.5%, *n* = 24), public services (12.7%, *n* = 21), nonessential healthcare services (e.g., physiotherapists (12%, *n* = 20)), and sciences (12%, *n* = 20). Employees reported an average organizational tenure of 11.16 years (SD = 10.43). The respondents’ average work week consisted of 34 h (SD = 9.88). The average age of the respondents was 43.91 years old (SD = 12.21); 47% of the respondents were male, and 53% were female. Finally, the respondents indicated that they were not worried about potential job loss, with an average rating of 2.5 (SD = 1.20) on a seven-point scale; however, this rating was slightly higher for employees conducting nonessential work (*M* = 2.70, *SD* = 1.20) than for employees conducting essential work (*M* = 2.27, *SD* = 1.17; Δ*M* = 0.433, *t* = 3.228, *df*=311, *p* = .001). None of the respondents in our sample lost their job during this study and reported work hours did not significantly differ between measurement occasions. We do not have information on the specific job settings or roles and how these may have been affected during the crisis.

### Measures

Table 1 presents all the measurement items with their corresponding factor loadings and standard errors for all respondents by essential versus nonessential work.

**Table 1:** Items and factor loadings

	Essential				Nonessential			
Items	R^2^	St. factor loading	Unst. factor loading	Se	R^2^	St. factor loading	Unst. factor loading	Se
Disruptions (T1)								
To what extent have your work procedures changed?	.36	.622	1.000 ^a^		.51	.712	1.000 ^a^	
To what extent have information-sharing mechanisms changed?	.25	.498	0.738	.14	.25	.502	0.704	.11
To what extent have the technologies used to complete work changed?	.45	.673	0.977	.12	.37	.604	0.691	.09
To what extent has the way you coordinate work changed?	.77	.878	1.306	.16	.74	.860	1.058	.10
To what extent has the way you organize work changed?	.89	.944	1.439	.17	.85	.924	1.218	.11
To what extent has the physical location of work changed?	.27	.519	1.075	.19	.21	.455	0.869	.16
Challenge stressors (T1)								
The amount of time I spend working	.67	.817	1.000 ^a^		.60	.774	1.000 ^a^	
The volume of work that must be accomplished in the allotted time	.65	.803	0.960	.05	.60	.773	1.113	.08
Time pressures I experience	.68	.824	1.063	.06	.72	.849	1.169	.09
The amount of responsibilities I have	.77	.878	1.110	.09	.61	.807	1.204	.11
The extent to which my job requires me to work hard	.86	.926	1.132	.08	.82	.906	1.221	.10
The number of complex and high-level skills my job requires	.81	.898	1.127	.08	.77	.867	1.203	.10
Hindrance stressors (T1)								
The degree to which politics rather than performance affects organizational decisions	.58	.760	1.000 ^a^		.45	.667	1.000 ^a^	
The inability to clearly understand what is expected of me on the job	.50	.708	0.779	.09	.56	.747	1.134	.13
The amount of red tape I need to go through to get my job done	.68	.826	1.044	.10	.61	.778	1.244	.14
The number of conflicting requests from two or more people	.73	.851	1.117	.10	.71	.841	1.450	.15
The amount of hassles to go through to get a project/assignment done	.74	.861	1.201	.11	.77	.879	1.547	.16
Social stressors—isolation (T1)								
I am separated from my coworkers.	.58	.759	1.000 ^a^		.68	.826	1.000 ^a^	
I often feel I am no longer close to anyone.	.58	.761	0.971	.11	.49	.696	0.811	.09
I am isolated from my coworkers.	.72	.847	1.183	.11	.73	.857	1.064	.09
I miss having coworkers around me.	.51	.716	0.915	.11	.41	.636	0.761	.09
Quality of change communication (T1)								
The communication my organization has provided …								
… has been useful.	.77	.878	1.000 ^a^		.63	.793	1.000 ^a^	
… has adequately answered my questions about the changes.	.73	.852	1.130	.08	.65	.809	1.203	.10
… has been positive.	.76	.873	0.974	.07	.71	.845	1.274	.10
… has been appropriate.	.88	.938	1.156	.07	.87	.931	1.279	.09
… has been timely.	.78	.883	1.162	.08	.72	.845	1.161	.09
… has been accurate	.72	.849	1.012	.07	.74	.862	1.165	.09
General distress (T2)								
Parcel 1 (MASQ-D30 items 4, 12, 13, 23)	.61	.781	1.000^a^		.69	.829	1.000^a^	
Parcel 2 (MASQ-D30 items 1, 17, 25)	.74	.862	1.218	.12	.83	.908	1.260	.09
Parcel 3 (MASQ-D30 items 7, 10, 28)	.74	.864	1.003	.09	.76	.870	0.983	.07

^a^The unit loading constraints used to scale the factors. Item numbers correspond with items reported in the original scale by Wardenaar et al. (2010)

*Disruption of work* was measured using six items adapted from Akgun et al. (2006). The original items were designed to assess changes in work routines during a team project. For this study, the measures were modified to reflect changes in work routines during the COVID-19 crisis. The respondents were asked to what extent aspects of their work had changed due to the COVID-19 regulations in the Netherlands. Respondents could answer on a seven-point Likert-type scale ranging from 1 = “not at all” to 7 = “changed completely.”

*Challenge stressors* were measured using six items adopted from Cavanaugh et al. (2000) and Rodell and Judge (2009). The challenge stressors referenced in the items included work-related demands or circumstances that are potentially stressful, including time pressure and amount of responsibility. The respondents were asked to indicate how stressful they felt the work-related items were using a seven-point scale ranging from “not at all stressful” (1) to “very stressful” (7).

*Hindrance stressors* were measured using five items adopted from Cavanaugh et al. (2000) and Rodell and Judge (2009). The hindrance stressors referenced in the items included perceived levels of red tape and role ambiguity. These demands are viewed as hindrance demands as they present obstacles to gains and growth (Rodell & Judge, 2009). Again, the respondents were asked to indicate how stressful they perceived the work-related items to be using a seven-point scale ranging from “not at all stressful” (1) to “very stressful” (7).

*Workplace isolation* was measured adopting four items from Marshall et al. (2007). We used the four items to evaluate the extent to which the individuals felt isolated from others (rather than the company). The responses were rated on a seven-point Likert-type scale ranging from 1 = “strongly disagree” to 7 = “strongly agree.”

*Quality of change communication* was measured using six items adapted from Bordia et al. (2004). The respondents were prompted to evaluate the communication they received from their employers during the COVID-19 crisis with the following instruction: “The following questions are about the communication your organization has provided throughout the COVID-19 crisis.” They were then asked to indicate their agreement with several aspects of the communication, including the usefulness, timeliness, accuracy, and adequacy. The respondents indicated their agreement on a seven-point scale ranging from “strongly disagree” (1) to “strongly agree” (7).

*General distress* was measured using the General Distress dimension of the Mood and Anxiety Symptom Questionnaire-D30 (MASQ-D30; Wardenaar et al., 2010). The general distress scale measures nonspecific symptoms of psychological distress or negative affect. General distress is a particularly relevant indicator of mental health during the COVID-19 pandemic as many factors—e.g., closure of work and schools, risk of infection, and reduced social contact—may contribute to the worsening of mental health in general and increased distress in particular (Daly & Robinson, 2020). The general distress subscale consists of 10 items including “I felt hopeless” and “I felt dissatisfied with everything.” Given that our sample is relatively small for structural equation modeling (SEM), in line with Corral-Frías et al. (2019) and Hau and Marsh (2004), we used three parcels. Parcel 1 consisted of “felt worthless,” “blamed myself for a lot of things,” “felt dissatisfied with everything,” and “felt inferior to others.” Parcel 2 was comprised of “felt confused,” “felt pessimistic about the future,” and “had trouble making decisions.” Finally, Parcel 3 consisted of “felt irritable,” “felt hopeless,” and “worried about a lot of things.” Employees were asked to rate the extent to which they experienced “feelings, sensations, problems and experiences that people sometimes have” in the past week on a 5-point Likert-type scale, with one being “not at all” and five being “extremely” (Wardenaar et al., 2010).

## Results

### Analysis

The hypothesized model and differences between the employees with essential and nonessential jobs were examined through multigroup SEM. To gauge the model fit, two incremental fit indices are examined: i.e., the Tucker-Lewis Index (TLI) and the Comparative Fit Index (CFI). Model fit indices of > .90 indicate a good model fit. Furthermore, two absolute fit indices are examined, a standardized version of the root mean squared residual (SRMR) and the root mean square of approximation (RMSEA), with cut-off values of ≤ 0.08 and ≤ 0.05, respectively, which indicate a close model fit (Hu & Bentler, 1999). In addition, the χ^2^ statistic is reported. Bootstrapping was used to obtain bias-corrected confidence intervals and standard errors for more parameters, including the (conditional) indirect effects (5000 bootstrap resamples were drawn from the data). In the analysis, we controlled for job security, age, and tenure, but, as these variables did not affect the relationships in the model, they were excluded from the final model. Although the sample size (N) to variable (p) ratio meets the criteria for SEM analysis, the N/p ratio is relatively small (Deng et al., 2018; Kline, 2011). We have replicated the findings using path modeling, to ensure the results were robust and the n/p ratio was not a problem. This analysis yielded similar results for all hypothesized relationships.

### Measurement Model

The measurement model demonstrated a good model fit: χ2 (772) = 1472.65; CFI = 0.90; TLI = 0.90; SRMR = 0.08; and RMSEA = 0.054 (CI: 0.049, 0.058). The average variance extracted (AVE) ranged between .49 and .77, indicating convergent validity. Discriminant validity was also examined through the maximum shared variance (MSV), which ranged between .12 and .63. Notably, the relatively high MSV between hindrance and challenge demands (.63) was in line with earlier studies. Additionally, the square root of the AVE was greater than the interconstruct correlations, indicating a good discriminant validity overall (see Table 2). Reliability was examined through the composite reliability (CR) and the maximum reliability (MaxR(H)), which ranged from .85 to .95 and from .86 to .96, respectively.

**Table 2:** Model validity statistics

Variable	M (SD)	CR	AVE	MSV	MaxR(H)	1	2	3	4	5	6
1. Disruption (T1)	5.23 (2.86)	.85	.49	.15	.90	**.70**					
2. Challenge stressor (T1)	2.84 (1.40)	.94	.74	.63	.95	.38	**.86**				
3. Hindrance stressor (T1)	2.65 (1.28)	.90	.65	.63	.91	.32	.80	**.80**			
4. Workplace isolation (T1)	4.39 (1.64)	.86	.60	.15	.86	.39	.16	.18	**.77**		
5. Communication quality (T1)	5.22 (1.15)	.95	.77	.12	.96	.04	−.13	−.35	−.02	**.88**	
6. General distress (T2)	17.10 (7.02)	.88	.70	.15	.88	.09	.19	.39	−.06	−.23	**.84**

*CR*, composite reliability; *AVE*, average variance extracted; *MSV*, maximum shared variance; *MaxR(H)*, maximum reliability. The square root of the AVE is reported on the diagonal. The correlations above .19 are significant at *p* < .05

### Descriptive Statistics

There were no significant differences in perceived disruptions between the employees conducting essential and nonessential work (*M*_*essential*_= 5.48, *SD* = 2.92; *M*_nonessential_= 5.01, *SD* = 2.80; Δ*M* = 0.476, *t* = 1.471, *df*=311, *p* = .143). Furthermore, the comparison of the stressors between the groups indicated that the employees conducting essential work experienced higher levels of hindrance stressors (*M* = 2.93, *SD* = 1.37) than their peers conducting nonessential work (*M* = 2.39, *SD* = 1.13; Δ*M* = 0.537, *t* = 5.087, *df*= 278.35, *p* < .001). A similar pattern emerged for the challenge stressors, as the employees with essential jobs reported higher mean challenge stressor scores (*M* = 3.25, *SD* = 1.50) than their peers with nonessential jobs (*M* = 2.47, *SD* = 1.19; Δ*M* = 0.786, *t* = 5.156, *df*=311, *p* < .001). In line with our assumptions, the employees conducting nonessential work reported higher levels of isolation (*M* = 4.61, *SD* = 1.58) than their peers conducting essential work (*M* = 4.14, *SD* = 1.67; Δ*M* = 0.469, *t* = 2.545, *df*=302.03, *p* = .011).

The mean scores on the general distress scale found in this study are relatively low compared to the reference values for general distress for the patient groups (M = 28.1, SD = 6.9) but higher than the means for the reference (nonpatient group) (M = 13.8, SD = 4.4) (Schulte-van Maaren et al., 2012). The scale scores are typically calculated as the sum of the items, as such scores can range from 10 to 50, with higher scores indicating more severe psychopathologies (Schulte-van Maaren et al., 2012); the respondents in our sample reported a mean score of 17.10 (SD = 7.02). There was no statistically significant difference between the employees conducting essential (*M* = 17.09, *SD* = 6.92) and nonessential work (*M* = 17.11, *SD* = 7.13; Δ*M* = 0.026, *t* = 0.033, *df*=311, *p* = .974).

Within the essential and nonessential groups, we further compared the means across the four biggest occupational groups represented in the data using analyses of variance (ANOVA) with Bonferroni corrections. For essential work, the sectors best represented in the data were health care, education, food supply, and essential governmental processes. For nonessential work, these sectors were healthcare, sciences, public and professional services. Across these industries, we found no significant differences in the hindrance or challenge stressors or the general distress measures for the essential or nonessential workers.

For essential work, we found no differences between the occupational groups in terms of the challenge or hindrance stressors and general distress. Regarding disruptions, the essential workers in education experienced greater disruptions compared those in health care (*M*_*education*_ = 5.00, *SD* = 1.35; *M*_*health care*_ = 3.87, *SD* = 1.12; Δ*M*_i-j_ 1.132, *p* < .001) and food supply (*M*_*health care*_ = 3.55, *SD* = 1.00; Δ*M*_i-j_ 1.455, *p* < .001). Moreover, the essential workers across these industries showed similar patterns in workplace isolation (*M*_*education*_ = 4.99, *SD* = 1.57; *M*_*health care*_ = 3.83, *SD* = 1.62; Δ*M*_i-j_ 1.166, *p* = .010) with those in food supply (*M*_*health care*_ = 3.40, *SD* = 1.66; Δ*M*_i-j_ 1.594, *p* = .005).

For the nonessential workers, we found only two differences: one in workplace isolation between the professional service employees and those from the science and higher education sectors (*M*_*science*_ = 5.68, *SD* = 1.14; *M*_*professional service*_= 4.46, *SD* = 2.00; Δ*M*_i-j_ 1.427, *p* = .029). In addition, the employees in the professional service sector experienced fewer disruptions than those in the science and higher education sectors did (*M*_*science*_ = 4.86, *SD* = 1.22; *M*_*professional service*_= 3.49, *SD* = 1.19; Δ*M*_i-j_ 1.372, *p* = .005).

### Structural Model

**Fig. 1:**
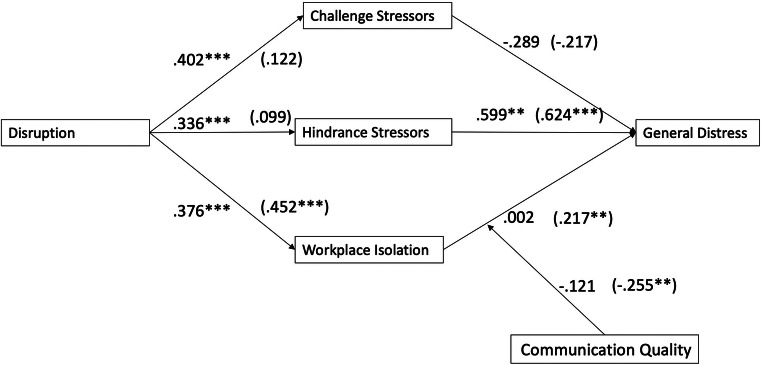
Simplified hypothesized model with standardized coefficients. Values in parentheses are the standardized coefficients for the employees in nonessential jobs. Significance is indicated as follows: ***p < .001, **p < .05. Only the significant interactions of workplace isolation and communication quality are shown

The structural model showed a good model fit: χ2 (776) = 1451.15; CFI = 0.91; TLI = 0.90; SRMR = 0.08; and RMSEA = 0.053 (CI: 0.049, 0.057). The difference between the unconstrained and constrained models (in which the paths were constrained to be equal for the employees with essential and nonessential jobs) was significant (Δ*Χ*^2^(77) = 144.28, *p* < 001). These findings suggested that essential or nonessential work qualified the model relationships; thus, we proceeded with testing the group differences for each hypothesis. Below, we report the unstandardized regression coefficients and confidence intervals; these are also summarized in Table 3. The standardized solutions are provided in Fig. 1. The model results demonstrated no significant relationship between work disruptions and general distress for the employees conducting essential (B_essential_ = –.011, 95% CI [–.060; .023], *p* = .459) or nonessential work (B_nonessential_ = .026, 95% CI [–.009; .065], *p* = .130).

**Table 3:** Unstandardized model parameters for hypotheses testing

	**Essential workers**					**Nonessential workers**			
			**Bootstrapping BC 95% CI**				**Bootstrapping BC 95% CI**		
	Result	B	Lower	Upper	*p*	B	Lower	Upper	*p*
*H1a* disruption → challenge stressors → general distress	Not supported	−.020	−.056	.002	.071	−.005	−.050	.003	.243
*H1b* disruption → hindrance stressors → general distress	Supported	.035	.011	.078	.004	.012	−.010	.062	.297
*H2* disruption → social stressors → general distress	Supported	.000	−.016	.011	.986	.019	.005	.042	.008
**Moderation of change communication quality**									
			**Full sample**						
			**Bootstrapping BC 95% CI**						
	Result	B	Lower	Upper	*p*				
*H3* disruption → challenge stressors → general distress	Not supported	.017	−.042	.076	.580				
*H4* disruption → hindrance stressors → general distress	Not supported	−.040	−.100	.020	.191				
*H5* disruption → social stressors → general distress	Supported	−.006	−.015	−.002	.034				

*BC*, bias corrected; *CI*, confidence interval; 5000 bootstrap samples

Hypothesis 1a reflected the assumption that work disruptions trigger challenge stressors, which may reduce general distress. We expected this relationship to be more profound for employees conducting essential work compared to those conducting nonessential work. The results indicated that work disruptions increased the challenge stressors for essential work (B_essential_ = .157, 95% CI [.083; .236], *p* < .001); however, these stressors did not significantly reduce general distress (B_essential_ = −.128, 95% CI [−.328; .031], *p* = .106), yielding a nonsignificant indirect relationship (B_essential_ = −.020, 95% CI [−.056; .002], *p* = .071). For the employees in nonessential jobs, the results did not demonstrate a significant relationship between the disruptions and challenge stressors, consistent with our hypothesis that this relationship would be significantly weaker for employees conducting nonessential work than for employees conducting essential work (B_nonessential_ = .037, 95% CI [−.024; .120], *p* = .239; *Ζ* = 2.892 *p* < .001). For this group, we also did not find support for the assumption that the challenge stressors reduce general distress (B_nonessential_ = −.135, 95% CI [−.573; .115], *p* = .305); we observed a nonsignificant indirect relationship (B_nonessential_ = −.005, 95% CI [−.050; .003], *p* = .243). Hence, although the results indicated significant differences between essential and nonessential work in the experience of challenge stressors triggered by work disruptions, the indirect effect hypothesis (H1a) was not supported.

Hypothesis 1b posited that work disruptions also trigger hindrance stressors, which increase general distress. We expected that this relationship would be more profound for those employed in essential than for those employed in nonessential work. The results indicated that the work disruptions were positively related to the hindrance stressors (B_essential_ = .122, 95% CI [.051; .206], *p* = .001). These hindrance stressors also demonstrated a positive relationship with general distress (B_essential_ = .285, 95% CI [.092; .537], *p* = .008). Hence, there was a significant indirect relationship between the disruptions and general distress through the hindrance stressors for the employees with essential jobs (B_essential_ = .035, 95% CI [.011; .078], *p* = .004). In contrast, the employees with nonessential jobs did not experience increased hindrance stressors due to the work disruptions (B_nonessential_ = .099, 95% CI [−.111; .335], *p* = .380). Again, in line with our hypothesis, this relationship was significantly weaker for the employees conducting nonessential than for those conducting essential work (*Ζ* = 2.594 *p* < .001). For the employees conducting nonessential work, the hindrance demands also increased general distress (B_nonessential_ = .624, 95% CI [.214; 1.198, *p* = .007). The indirect relationship between the work disruptions and general distress through the hindrance demands for the employees engaged in nonessential work was nonsignificant (B_nonessential_ = .012, 95% CI [−.010; .062], *p* = .297). Overall, these results supported the reasoning reflected in Hypothesis 1b.

Hypothesis 2 concerned workers’ feelings of isolation from colleagues. Specifically, we assumed that work disruptions trigger social isolation, which increases general distress. We suspected that this relationship would be more profound for employees with nonessential jobs than for those with essential jobs. For the employees in essential jobs, the results demonstrated that the work disruptions increased isolation (B_essential_ = .376, 95% CI [.213; .524], *p* < .001); however, isolation did not significantly affect general distress for the employees conducting essential work (B_essential_ = .002, 95% CI [−.198; .190], *p* = .986). Hence, the indirect relationship between the disruptions in general distress through isolation was not significant for the employees with essential jobs (B_essential_ = .000, 95% CI [−.016; .011], *p* = .989). For the employees with nonessential jobs, the findings also showed a significant positive relationship between the work disruptions and isolation (B_nonessential_ = .233, 95% CI [.147; .345], *p* < .001) and between isolation and general distress (B_nonessential_ = .081, 95% CI [.023; .151], *p* = .009). Notably, this effect was significantly stronger for the employees with nonessential jobs than for those with essential jobs (*Ζ* = 1.965, *p* < .05). Hence, a significant indirect effect between disruption and general distress through isolation was found for the employees conducting nonessential work (B_nonessential_ = .019, 95% CI [.005; .042], *p* = .008). These findings supported the reasoning reflected in Hypothesis 2.

### Moderating Effect

#### Moderating Effects

Hypotheses 3–5 posited that communication quality may aid in mitigating the negative implications of work and social stressors for general distress. As we do not hypothesize differences across groups for the moderation effect of communication quality, moderations were tested for the full sample. We found that the interactions of communication quality with the challenge stressors (B = .017, 95% CI [−.042; .076] *p* = .580) and hindrance stressors (B= −.040, 95% CI [−.100; .020] *p* = .191) were not significant. These findings do not provide support for Hypotheses 3 and 4. The findings demonstrate a significant moderation effect between social isolation and communication quality on general distress (B= −.006, 95% CI [−.015; −.002] *p* = .034). This suggests that the positive relationship between social isolation and general distress is mitigated by communication quality, such that higher perceived communication quality reduces the impact of isolation on stress. These findings support Hypothesis 5. Finally, we conducted a post hoc analysis to further inspected the interaction effects across occupational groups. Notably, the interactions of communication quality with the challenge stressors (B_nonessential_ = .048, *p* = .290; B_essential_ = −.012, *p* = .682) and hindrance stressors (B_nonessential_ = −.036, *p* = .480; B_essential_ = −.024, *p* = .380) were not significant for either group. However, interestingly, employees in nonessential jobs seem to benefit from change communication quality to mitigate the impact of social isolation (B_nonessential_ = −.013, 95% CI [−.029; −.003], *p* = .005) compared to employees in essential jobs (B_essential_ = −.006, 95% CI [−.022; .004], *p* = .198); as we did not observe the interaction for this group of workers.

## Discussion

The findings demonstrate that employees conducting essential and nonessential work both experience general distress as a result of workplace disruptions. However, for employees with essential jobs, general distress is predominantly qualified by the work stressors that these disruptions induce. The underlying mechanism for employees with nonessential jobs clearly demonstrates the importance of social stressors—here, social isolation. The remote work mandate has led employees conducting nonessential work to feel more isolated, which has affected their mental health, specifically distress. Interestingly, our findings also demonstrate that employees conducting essential work feel more isolated as a result of the experienced disruptions during the outbreak period. This finding makes sense, as social distancing is also practiced in the workplace to ensure the safety of workers, clients, patients, and customers. Notably, although the values for general distress were relatively low in the early stages of the COVID-19 crisis, indicating employees were coping relatively well, employees’ mental health may have worsened as work and social stressors prevailed throughout the crisis. The findings inform which underlying stressors contributed to the worsening of mental health across occupational groups. Finally, we demonstrate that the quality of organizational communication about the crisis helps to mitigate the negative impact of isolation on general distress, but only for employees conducting nonessential work.

### Theoretical Implications

The present study is important because it directs attention to the consideration of how various employees and occupational groups may appraise workplace stressors differently in response to disruptions and transitions to remote work. Specifically, the study has at least two important theoretical implications. First, this study broadens the scope of research on the challenge stressor-hindrance stressor framework by moving beyond individual and organizational levels of analysis to include (molar) environmental triggers (Morgeson et al., 2015). Conversely, Johns (2006) describes this as the “omnibus context,” which refers to the who, what, when, where, and why of an event. The *when* heuristic refers to the time at which the research was conducted or the research event occurred, while the *who* heuristic refers to the occupational and demographic contexts. During the COVID-19 pandemic (“when”), occupational groups were categorized as essential or nonessential (“who”). This pandemic vividly demonstrated how extreme events may reshape the perceptions of individuals and organizations about work and occupations and result in both micro and macro shifts in the world of work (Carnevale & Hatak, 2020; Kramer & Kramer, 2020). Subsequently, Johns (2006) theorized that discrete contextual variables—in the context of this study, work, and social stressors—provide the explanatory link between more descriptive and general omnibus contexts and work outcomes. Because workers are categorized as essential or nonessential (omnibus context), we assumed these groups would experience general distress through different underlying mechanisms (discrete context). The findings largely provide empirical support for these assumptions, thereby advancing our understanding of how molar environmental triggers may impact individual perceptions of work across organizational groups. In doing so, we connect with and extend previous work that points toward the importance of contextual factors in the context of challenge and hindrance stressors and their effects (Bakker & Sanz-Vergel, 2013; Naseer et al., 2020; Webster et al., 2011). To our knowledge, this is the first study to demonstrate the differential effects of work and social stressors on mental health for two different occupational groups.

Second, the findings suggest that the inclusion of social stressors in the challenge stressor-hindrance stressor framework may be valuable in understanding individual health outcomes (Dormann & Zapf, 2004; Harris et al., 2009). Especially in the context of renewed interest in remote and virtual work practices, individuals will increasingly work in dispersed locations (Raghuram et al., 2019; Rudolph et al., 2020). However, the findings also demonstrate that employees with essential jobs experience isolation in the workplace as social-distancing measures extend there as well. Indeed, sociologists, psychologists, and management scholars have expressed concerns about the discourse around social-distancing practices (Gibson, 2020; Menjivar et al., 2020). Recently, against the backdrop of the COVID-19 pandemic, Gibson (2020) called upon organizational scholars to embrace “care in connecting” as a central research theme, suggesting that social aspects of work—i.e., inclusions, copresence, and vitality—may serve as an “antidote to prejudice, isolation, and hopelessness” (p. 4). Our findings present evidence that the disruptions triggered by the pandemic not only increase social stressors but also increase general distress for employees conducting nonessential work. In that sense, research on the challenge stressor-hindrance stressor framework should adapt to current organizational realities that are increasingly promoting telework, remote work, or virtual work arrangements. Research in these areas has demonstrated that not only work stressors such as communication or work overload (Nurmi, 2010) but also social aspects related to trust and social exchange play an important role in team effectiveness (Breuer et al., 2016).

Importantly, the results also demonstrate that isolation is a concern not only to those working in physically isolated places. Indeed, in line with recent criticism on “geographical determinism” (O’Leary et al., 2014, p. 1221), our findings imply that employees attach similar symbolic meanings of isolation to their separation from colleagues whether they are isolated due to working in remote locations or due to working under six-foot social-distancing workplace regulations. However, the consequences of isolation across these conditions for distress may be different. In summary, our findings advance the understanding of the ways in which different occupational groups may be differently affected by the crisis (Kramer & Kramer, 2020; Spurk & Straub, 2020) by demonstrating the diverse role of work and social stressors underlying psychological distress across occupational groups.

### Practical Implications

The findings suggest that the quality of change communication does not mitigate the relationship between work stressors and general distress. However, for employees conducting nonessential work, change communication quality is important, as it reduces the positive relationship between social isolation and general distress. Thus, employees in nonessential jobs who feel isolated may find some comfort and clarity through high-quality change communication, which could reduce the negative implications of social isolation on general distress.

However, the findings also suggest that employees conducting essential work might need more than high-quality communication if they are to continue their essential work in the face of surging work stressors imposed by the crisis. Our findings are partially aligned with previous research that demonstrated that organizational support might help employees effectively meet the demands of challenge stressors but does not help them cope with hindrance stressors (Wallace et al., 2009). Hence, organizations may need to provide additional resources to help employees with essential jobs deal with hindrance stressors. Rodell and Judge (2009) suggested that organizations have many tools to manage hindrance stressors, including the ability to minimize unnecessary paperwork or provide adequate resources and materials. Especially for employees conducting essential work during the COVID-19 pandemic, adequate resources and safety materials are crucial for facilitating healthy, safe, and decent work conditions.

In addition to making efforts to reduce work stressors for employees conducting essential work, organizations employing these workers may consider mindfulness as a universal intervention. In other extreme work environments characterized by high-demand, high-stress occupational settings, e.g., the military, mindfulness strategies have been shown to enhance workers’ ability to tolerate adversity and to facilitate healthy engagement with potentially traumatic stressors (Smith et al., 2011). Mindfulness can be considered a selective strategy to improve emotional regulation and self-regulatory control that reduces harmful health-related behaviors such as risk-taking (Nassif et al., 2019). Hence, mindfulness strategies may help workers cope with extremely stressful work environments.

### Limitations and Future Research

We recognize that our research has a number of limitations. First, we used a 3-week time interval between measuring the predictors and the outcome, which was sufficient to eliminate most of the concurrent correlation (Ostroff et al., 2002). However, a complete three-wave panel design would have allowed us to test the proposed indirect effects; make stronger claims about the causality of the relationships; and detect differences in the respondents’ perceived disruptions, stressors, and general distress as the crisis unfolded.

Second, this study focused on general distress. Although general distress is an important indicator of mental health, future studies could consider a broader range of mental health outcomes including anxiety, depression, or overall life satisfaction. Future studies may further investigate how disruptions may affect different mental health symptoms, e.g., depression and anxiety. In addition, the results indicate that employees experience a moderate amount of distress (in the initial stages of the pandemic). It seems worthwhile to consider how employees’ mental health has been continuously affected as stressors and demands prevail throughout the COVID-19 crisis.

Third, this study investigated the impact of work disruption during the COVID-19 pandemic for employees with essential jobs and those with nonessential jobs with temporary or permanent contracts. Although this distinction is important and media and policy makers have made this distinction a focal point in the attempts to contain the pandemic, an investigation of other work arrangements was outside the scope of the study. These groups include agency workers, gig workers, small business owners, and self-employed workers (Spurk & Straub, 2020). Future studies may examine how different work arrangements influence work and social stressors, as well as how these stressors impact work outcomes. In addition, essential and nonessential workers are unequally distributed across occupational groups, as, for instance, healthcare has a higher proportion of essential workers, than, for instance, the professional or financial service sector. Future research could pursue an even finer grained understanding of the relationship between disruptions and general distress by considering how differences between occupational groups inform these results.

Fourth, our sample was relatively small, and the survey data did not provide an in-depth understanding of the work conditions of our respondents. This limitation was in part a consequence of the research design, as we obtained samples of employees with essential and nonessential jobs but did not limit the sampling based on specific organizational contexts or job content. In addition, we do not have information on the extent to which specific job roles have changed or the extent to which nonessential workers could ignore remote work instructions. Future studies might examine how the nature of work may impact the meaning that employees attach to the disruptions and stressors they experience. The present study contributes to an understanding of the impact of work disruptions caused by regulations to contain the virus on general distress for employees conducting essential and nonessential work.

## Footnotes

1 https://www.government.nl/topics/coronavirus-covid-19/childcare-for-children-of-people-working-in-crucial-sectors

2 https://www.cbs.nl/en-gb/dossier/coronavirus-crisis-cbs-figures/economic-impact-of-covid-19

3 https://www.nrc.nl/nieuws/2020/06/03/willekeur-van-coronaregels-ondermijnt-het-vertrouwen-a4001597

4 https://www.government.nl/documents/publications/2020/03/20/childcare-for-children-of-people-working-in-crucial-sectors
